# Unilateral digital clubbing in complex regional pain syndrome after brachial plexus injury

**DOI:** 10.1093/omcr/omad101

**Published:** 2023-09-25

**Authors:** A Armas-Salazar, F X Cid-Rodríguez, E Abarca-Rojano, J D Carrillo-Ruiz

**Affiliations:** Functional & Stereotactic Neurosurgery & Radiosurgery Service, General Hospital of México, Mexico City, Mexico; Postgraduate Department, School of Higher Education in Medicine, National Polytechnic Institute, Mexico City, Mexico; Functional & Stereotactic Neurosurgery & Radiosurgery Service, General Hospital of México, Mexico City, Mexico; Postgraduate Department, School of Higher Education in Medicine, National Polytechnic Institute, Mexico City, Mexico; Scholarship Holder of the Direction of Quality and Education on Health, Secretary of Health, Mexico City, Mexico; Postgraduate Department, School of Higher Education in Medicine, National Polytechnic Institute, Mexico City, Mexico; Functional & Stereotactic Neurosurgery & Radiosurgery Service, General Hospital of México, Mexico City, Mexico; Faculty of Health Sciences Direction of Anahuac University, Mexico City, Mexico; Neuroscience Coordination, Psychology Faculty, Anahuac University Mexico, Mexico City, Mexico

## CLINICAL IMAGE DESCRIPTION

**Figure 1:**
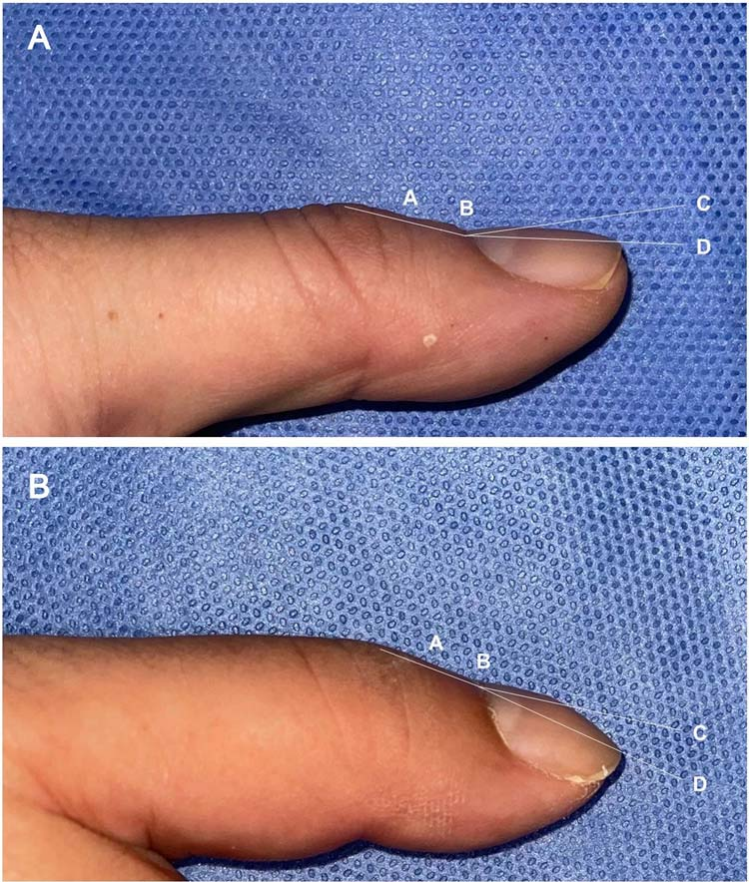
Patient with unilateral digital clubbing in complex regional pain syndrome after brachial plexus injury (6 months of follow-up after injury). Representation of the nail angles (**A**, **B**). ‘A’: Maximum depression in the distal interphalangeal joint. ‘B’: Nail-phalangeal angle (Lovibond). ‘C’: Nail plate. ‘D’: Hyponychial angle. A. Left thumb (normal (<180°)). B. Right thumb (Affected (>180°)).

A 38-year-old male patient was evaluated after three months of being hit by vehicle, which caused a non-penetrating right brachial plexus injury (BPI) (C5-T1) with total avulsion. The patient presented complete lack of mobility and sensation in the extremity, as well as neuropathic pain of high intensity (10 according to visual analogue scale (VAS)), with repercussions on mood and sleep disturbances. MRI images showed an hypointense lesion located in the right brachial plexus from C5-T1, electromyography showed pre-ganglionic right panplexopathy with axonotmesis-type lesion and nerve conduction block. These findings integrate a complex regional pain syndrome (CRPS) type 2 [1], pharmacological management with maximum doses of tramadol and gabapentin were used, at the same time consultation with physical therapy and mental health were employed as non-pharmacological management, both showed non-response and lead to surgical management (surgical neurolysis). Clinical evaluation at one month after the surgery showed improvement of pain from 10 to 4 according to VAS. An incidental finding of unilateral digital clubbing was made by comparing both hands and measuring the angles on the nails. The Levibond angle on the nails was increased of the right hand, as well as the presence of autonomic changes [2] (Fig. 1).

The relevance of this finding beyond the rarity is to highlight a mechanism that could be associated with pain origin in the CPRS and is going unnoticed. In the context of CPRS management, this usually focuses on controlling the inflammatory components. However, it has been observed that during the chronic phase, the prolonged release of endothelin-1 results in an excessive outflow from the sympathetic nervous system, culminating in vasoconstriction (hypoxia), a mechanism that could explain the development of clubbing hands secondary to hypoperfusion. For this reason, we believe that the use of sympathetic blocks should be further explored in these cases [3].

## CONFLICT OF INTEREST STATEMENT

The authors have no conflicts of interest to declare.

## FUNDING

This study had no funding sources.

## ETHICAL APPROVAL

No approval is required.

## CONSENT

The patient signs a written consent for the publish of this material.

## GUARANTOR

This study had no guarantors.

## DATA AVAILABILITY

Data available with a reasonable request.
